# In Vitro and In Vivo Studies on* Quercus acuta* Thunb. (Fagaceae) Extract: Active Constituents, Serum Uric Acid Suppression, and Xanthine Oxidase Inhibitory Activity

**DOI:** 10.1155/2017/4097195

**Published:** 2017-03-22

**Authors:** In-Soo Yoon, Dae-Hun Park, Min-Suk Bae, Deuk-Sil Oh, Nan-Hui Kwon, Jung-Eun Kim, Chul-Yung Choi, Seung-Sik Cho

**Affiliations:** ^1^College of Pharmacy, Pusan National University, Geumjeong, Busan 46241, Republic of Korea; ^2^Department of Oriental Medicine Materials, Dongshin University, Naju, Jeonnam 58245, Republic of Korea; ^3^Department of Environmental Engineering, Mokpo National University, Muan, Jeonnam 58554, Republic of Korea; ^4^Jeollanamdo Wando Arboretum, Wando, Jeonnam 59105, Republic of Korea; ^5^Department of Pharmacy, College of Pharmacy, Mokpo National University, Muan, Jeonnam 58554, Republic of Korea; ^6^Jeonnam Bioindustry Foundation, Institute of Natural Resources Research, Jangheung, Jeonnam 59338, Republic of Korea

## Abstract

*Quercus acuta* Thunb. (Fagaceae) (QA) is cultivated as a dietary and ornamental plant in China, Japan, South Korea, and Taiwan. It has been widely used as the main ingredient of acorn tofu, a traditional food in China and South Korea. The aim of this study was to determine in vitro and in vivo xanthine oxidase (XO) inhibitory and antihyperuricemic activities of an ethyl acetate extract of QA leaf (QALE) and identify its active phytochemicals using gas chromatography-mass spectrometry (GC-MS) and liquid chromatography (LC) systems. The QALE was found to possess potent in vitro antioxidant and XO inhibitory activities. In vivo study using hyperuricemic mice induced with potassium oxonate demonstrated that the QALE could inhibit hepatic XO activity at a relatively low oral dose (50 mg/kg) and significantly alleviate hyperuricemia to a similar extent as allopurinol. Several active compounds including vitamin E known to possess XO inhibitory activity were identified from the QALE. To the best of our knowledge, this is the first study that reports the active constituents and antihyperuricemic effect of QA, suggesting that it is feasible to use QALE as a food therapy or alternative medicine for alleviating hyperuricemia and gout.

## 1. Introduction

Hyperuricemia is a condition with an abnormally high concentration of uric acid in the blood. It can be caused by regular intake of purine-rich food and invariably accompanied by gout, chronic kidney disease, and/or other metabolic syndrome [1]. Xanthine oxidase (XO) catalyzes the oxidation of hypoxanthine and xanthine to uric acid. It can generate reactive oxygen species (ROS) [2]. Uric acid, the final oxidation product of purine metabolism, is eliminated mainly via urinary excretion from the kidney. XO-mediated overproduction of uric acid can lead to hyperuricemia, a major cause of gout. Hyperuricemia is associated with increased risk of cardiovascular disorder, nephrolithiasis, and diabetes [3]. Gout is a metabolic disorder characterized by marked hyperuricemia with the deposition of urate monohydrate crystals in joints and kidneys, resulting in gouty arthritis and uric acid nephrolithiasis [4]. It has been reported that one million people in the United States and 0.3 million people in South Korea suffer from gouty arthritis [5]. Moreover, XO-mediated excessive production of ROS can increase the level of oxidative stress, resulting in several pathological processes such as inflammation, atherosclerosis, cancer, and aging [6, 7]. Thus, monitoring the level of XO is often an important part in the prevention and treatment for hyperuricemia and other diseases caused by oxidative stress.

One of the therapeutic approaches to treat and prevent hyperuricemia is to use XO inhibitors that can block the final step of XO reaction to synthesize uric acid, thus reducing the levels of uric acid. Allopurinol is the only XO inhibitor approved for clinical use. However, it can cause several side effects such as hypersensitivity reactions, nephropathy, fetal liver necrosis, and enhancement of 6-mercaptopurine toxicity [8, 9]. Therefore, it is important and necessary to search for safe herbal preparations and phytochemicals that are effective for hyperuricemia and gout [10, 11]. In the “Dongui Bogam” which is regarded as one of the most important classics of oriental medicine today, three plants (i.e.,* Carthamus tinctorius*,* Prunus persica*, and* Angelica gigas*) were reported to be effective for the prevention and anesis of gout (Dongui Bogam: Japbyeongpyeon, Hanbulhakyesa, Seoul, Republic of Korea, 2016). Therefore, we preliminarily screened in vitro xanthine oxidase (XO) inhibitory activity of extracts from 229 dietary and/or medicinal plants including the three plants mentioned above. However, unfortunately, the XO inhibitory activity of the three plant extracts was not so high, while, unexpectedly, an extract from the leaf of* Quercus acuta* Thunb. (Fagaceae) (QA) showed profoundly higher activity compared to the other plant extracts (data not shown). Based on these preliminary results, we designed the present study on QA, which could contribute to developing a novel complementary and alternative medicine for treating hyperuricemia or gout.

QA is cultivated as a dietary and ornamental plant in China, Japan, South Korea, and Taiwan [12]. The fruit of QA (acorn) has been widely used as the main ingredient of acorn tofu, a traditional food in China and South Korea [13]. To date, only one research paper has been found in the literature with two compounds identified from the trunk of QA. The two compounds have been reported to possess antimicrobial properties [12]. However, to the best of our knowledge, there have been no published reports on the active constituents and biological activity of extract of QA leaf (QALE).

Therefore, the objective of this study was to determine antioxidant, XO inhibitory activity, and antihyperuricemic activities of the QALE and identify its major active phytochemicals. Gas chromatography-mass spectrometry (GC-MS) and liquid chromatography analyses were performed for chemical profiling. In vitro DPPH free radical assay was used to determine antioxidant activity. XO inhibitory and antihyperuricemic activities were assessed in potassium oxonate-induced hyperuricemic mice.

## 2. Materials and Methods

### 2.1. Plant Materials

QA leaves were collected from the Wando Arboretum (Wando, Republic of Korea) and identified by Dr. Deuk-Sil Oh affiliated to the Wando Arboretum. A voucher specimen (MNUCSS-QA-01) was deposited at Mokpo National University (Muan, Republic of Korea). Air-dried and powdered QA leaves (10 g) were extracted twice with ethyl acetate (100 mL) at room temperature for 3 days. The resultant ethyl acetate solution was evaporated, dried, and stored at −50°C. It was used for further in vitro and in vivo experiments.

### 2.2. Animals

Male ICR mice (4-weeks old) were purchased from Orient Bio, Co. (Seongnam, Republic of Korea). They were retained in a clean room at a temperature of 20–23°C with 12 h light (07:00–19:00) and dark (19:00–07:00) cycles at relative humidity of 50 ± 5%. Mice were housed in ventilated mice cages (Tecniplast USA, Inc.) with filtered and pathogen-free air. Food (Agribrands Purina Korea, Inc.) and water were provided ad libitum. All animal experiments were carried out in accordance with the Guidelines of the Animal Investigation Committee of Jeonnam Bioindustry Foundation (Naju, Republic of Korea) with approval number of JINR1517.

### 2.3. Chemical Profiling by GC-MS Analysis

GC-MS analysis was conducted using a previously reported method with slight modifications [14]. Briefly, Agilent 7890 gas chromatograph system was used to analyze scanned organic compound. It was coupled to a quadrupole Agilent 5975C electron ionization (70 eV) mass spectrometric detector (Agilent Technologies, Palo Alto, CA, USA) equipped with a Agilent HP-5MS fused silica capillary column (30 mm* l.* × 0.25 mm* i.d.*, 0.25-*μ*m film thickness). GC-MS was tuned using perfluorotributylamine (PFTBA) with mass fragments of 69.0, 219.0, and 502.0* m/z* under electron ionization (EI) conditions. GC oven was heated using the following program: isothermal at 65°C for 10 min and 10 min^−1^ to 300 with helium (He) as carrier gas. Transfer line was heated at 300°C. The mass spectrometer was operated in scan mode of 50–550 amu. All mass spectra were compared to the data system library (NIST 2008). The operation parameters for the GC were shown in Table 1.

### 2.4. Constituents Profiling by LC Analysis

Constituent profiling of QALE was performed with HPLC. Previously, we purified and identified the three minor constituents, namely, quercetin, luteolin, and apigenin using column chromatography and preparative thin layer chromatography. All HPLC analyses were performed using Alliance 2695 HPLC system (Waters, Millford, MA, USA) equipped with a photodiode array detector. Agilent Zorbax extended C18 (5 *μ*m, 150 mm* l.* × 5 mm* i.d.*) analytical column was used with a mobile phase consisting of solvent A (acetonitrile) and solvent B (water containing 0.2% phosphoric acid). Gradient elution (from 10/90 to 100/0, v/v) was performed at flow rate of 1.0 mL/min (Table 1). Column temperature was maintained at 25°C. The detection wavelength was set at 270 nm for quercetin, luteolin, and apigenin. Solvent was filtered through 0.22 *μ*m filter and degassed. The sample injection volume was 10 *μ*L.

### 2.5. DPPH Free Radical Assay

Antioxidant activity of QALE was determined using 2,2-diphenyl-1-picrylhydrazyl (DPPH) radical scavenging assay. DPPH radicals with the maximum absorbance at 517 nm will disappear when they are reduced by antioxidant compound. QALE solution (1 mL) containing 1 to 20 mg of QALE was added to 0.4 mM DPPH QALE solution (1 mL) and mixed. The mixture was allowed to react at room temperature in the dark for 10 min. Absorbance value at 517 nm was measured using a microplate reader (Perkin Elmer, Waltham, MA, USA). The radical scavenging activity was calculated as a percentage using the following equation:

(1) DPPH  radical  scavenging  activity  %  =  1−AsampleAblank×100.

The DPPH free radical scavenging activities of samples were compared in terms of their IC_50_ (*μ*g/mL) values [15].

The reducing power of QALE was determined using a modified reducing power assay method. The sample (0.1 mL) was added to 0.2 M sodium phosphate buffer (0.5 mL) and 1% potassium ferricyanide (0.5 mL) followed by incubation at 50°C for 20 min. After the incubation, 10% trichloroacetic acid solution (0.5 mL) was added to the reaction mixture followed by centrifugation at 12000 rpm for 10 min. The supernatant was mixed with distilled water (0.5 mL) and 0.1% iron (III) chloride solution (0.1 mL). Absorbance value of the resulting solution was measured at 700 nm. Reducing powers of samples were expressed as vitamin C equivalents [15].

### 2.6. Determination of Total Phenolic Content

The total phenolic content was determined using Folin-Ciocalteu assay [15]. Water solution (1 mL) containing 5 mg of QALE or standard was mixed with 1 mL of 2% sodium carbonate solution and 1 mL of 10% Folin-Ciocalteu's phenol reagent. After incubating the mixture at room temperature for 10 min, its absorbance was measured at 750 nm using a microplate reader (Perkin Elmer) and compared to the calibration curve of gallic acid. Results were expressed as milligrams of gallic acid equivalents per gram of sample [15].

### 2.7. Determination of Total Flavonoids

Total flavonoid content was determined with previously reported colorimetric method [15]. Briefly, 0.5 mL of sample solution was mixed with distilled water (2 mL) and 5% NaNO_2_ solution (0.15 mL). After incubation for 5 min, 0.15 mL of 10% AlCl_3_ solution was added to the mixture. After incubation at room temperature for 5 min, 4% NaOH solution (2 mL) was added to the mixture. After bringing the final volume to 5 mL with water, the mixture was thoroughly mixed and allowed to stand at room temperature for 15 min. The absorbance value of the resultant mixture was measured at 415 nm. Total flavonoid content was expressed as amount (mg) of flavonoid (quercetin) per amount of extract (g).

### 2.8. Determination of In Vitro Xanthine Oxidase (XO) Inhibitory Activity

XO inhibitory activity was measured by monitoring uric acid formation in xanthine oxidase system as described previously [16]. The assay system consisted of 0.6 mL phosphate buffer (100 mM; pH 7.4), 0.1 mL sample, 0.1 mL XO (0.2 U/mL), and 0.2 mL xanthine (1 mM; dissolved in 0.1 N NaOH). The reaction was initiated by adding the enzyme with or without inhibitors. Changes in absorbance of the mixture at 290 nm for 15 min compared to the absorbance of reagent blank were determined. A 0.2 mL aliquot of 1 N HCl was used to stop the enzymatic reaction. Allopurinol was used as positive control.

### 2.9. Pretreatment and Hyperuricemia Induction in Mice

The QALE or ALP was dissolved in 0.3% CMC-Na aqueous solution. Five groups of mice (*n* = 5 for each group) were pretreated once daily for 7 days as follows: mice in two negative control groups (Nor and HU groups) received 0.3% CMC-Na aqueous solution; mice in the positive control group (ALP group) received ALP solution at a dose of 10 mg/kg; mice in QA50 and QA250 groups received the QALE solution at doses of 50 and 250 mg/kg, respectively. Hyperuricemia was induced in mice by potassium oxonate, a uricase inhibitor as described previously [17]. Potassium oxonate (dissolved in PBS; 250 mg/kg) was administered to all mice except those in the Nor group intraperitoneally, 1 h before the last pretreatment on the 7th day (mice in the Nor group received PBS instead of potassium oxonate). Finally, 1 h after the last pretreatment on the 7th day, approximately 0.5 mL blood samples were collected via the tail vein, allowed to clot for 1 h at 4°C, and centrifuged at 10000*g* for 15 min to obtain serum. The resultant serum samples were stored at −80°C until further analysis.

### 2.10. Determination of In Vivo Uric Acid Concentration and XO Activity

Serum uric acid concentration was measured using standard diagnostic kits (Abcam; Cambridge, UK). Each assay was performed in triplicate. The residual activity of XO in mouse liver and serum were spectrophotometrically determined by monitoring uric acid formation from xanthine [18]. Mice livers (0.5 g) were homogenized in 1 mL of 50 mM sodium phosphate buffer (pH 7.4). The homogenates were centrifuged at 3000*g* for 10 min at 4°C. After removing lipid layer, supernatant was centrifuged at 10000*g* for 60 min at 4°C. The resultant supernatant was used to determine XO residual activity and total protein concentration. A 0.12 mL aliquot of xanthine solution (250 mM) was added to a test tube containing 10 *μ*L liver homogenate and 0.54 mL potassium oxonate solution (1 mM) in 50-mM sodium phosphate buffer (pH 7.4) that was previously incubated at 35°C for 15 min. The reaction was stopped after 0 and 30 min of reaction by adding 0.1 mL of 0.6 M HCl. Thereafter, the test tube was centrifuged at 3000*g* for 5 min. The absorbance value of the supernatant was measured at 295 nm using a UV/VIS spectrophotometer. Total protein concentration was determined spectrophotometrically with Bradford method [19]. XO activity was expressed as micromoles of uric acid formed per minute (U) per milligram protein.

### 2.11. Statistical Analysis

All data were expressed as mean ± standard deviation and rounded to have three significant figures. For difference between two means for unpaired data,* t*-test was performed. For difference among three or more means for unpaired data, analysis of variance (post hoc test: Tukey*ʼ*s multiple range test) was performed. A *p* value less than 0.05 was considered to be statistically significant.

## 3. Results

### 3.1. In Vitro Antioxidant Activity

The in vitro antioxidant activity of QALE was determined with the DPPH and reducing power assays. The DPPH free radical scavenging assay is a widely used method to evaluate free radical scavenging ability of plant extracts. As a result, a relatively low IC_50_ value (10.6 ± 0.5 *μ*g/mL) of QALE was observed in the DPPH radical scavenging assay (Table 2). To determine the reducing capability of QALE, the transformation of Fe^3+^ to Fe^2+^ was measured in the presence of the extract. The reducing power of 50-*μ*g QALE was determined to be equivalent to that of a relatively high amount of ascorbic acid (36.3 ± 3.5 *μ*g).

### 3.2. Total Phenolic and Flavonoid Contents

Total phenolic content of QALE was measured with gallic acid equivalence method using Folin-Ciocalteu's reagent (standard curve equation:* y* = 0.023*x* + 0.124, *r*^2^ = 0.999) [15]. The total phenolic content of QALE was determined to be 126 ± 2 mg/g eq. gallic acid (Table 2). The total flavonoid content of QALE was measured with quercetin equivalence method (standard curve equation:* y* = 6.694*x* + 0.035, *r*^2^ = 0.999). As shown in Table 2, total flavonoid content of QALE was determined to be 27.6 ± 1.4 mg/g eq. quercetin.

### 3.3. In Vitro XO Inhibitory Activity

**Figure 1:**
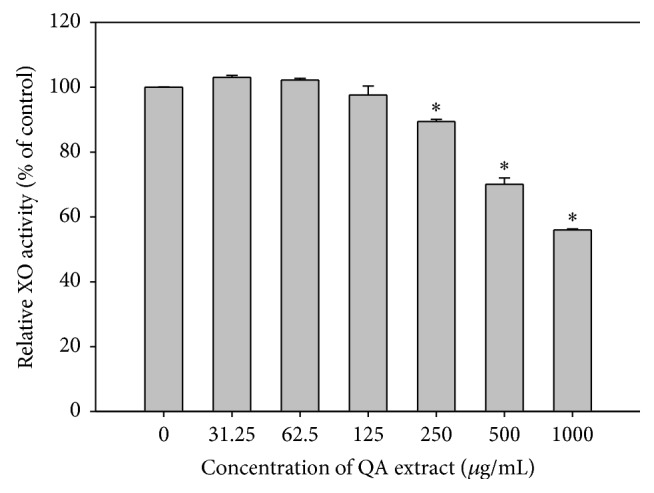
In vitro xanthine oxidase (XO) inhibitory activity of QALE at various concentrations ranging from 0 to 1000 *μ*g/mL; ^*∗*^significantly different from the control group (QALE at a concentration of 0 *μ*g/mL). The rectangular bars and their error bars represent the means and standard deviations, respectively (*n* = 5).

The XO inhibitory activity of QALE was expressed as a suppression rate of uric acid production. Allopurinol (positive control) at concentration of 30 *μ*g/mL significantly inhibited the activity of XO (by 64.55 ± 2.55%). Results of the in vitro XO inhibitory activity of QALE are shown in Figure 1. The relative XO activity of QALE at concentrations of 250 *μ*g/mL or higher was significantly higher than that of control group (without QALE) in a dose-dependent manner. The QALE at concentration of 1 mg/mL showed the highest XO inhibitory activity (by 44.0 ± 0.3%).

### 3.4. In Vivo Antihyperuricemic Effect of QALE on Serum Uric Acid Levels

**Figure 2:**
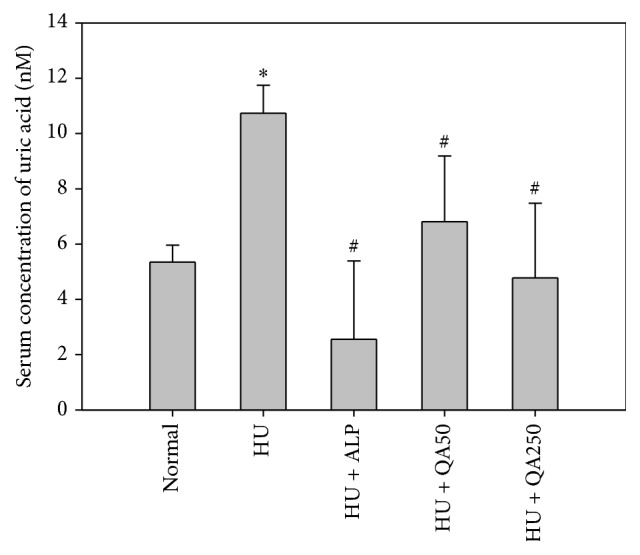
Serum uric acid levels after oral administration of saline in normal mice (normal) or after oral administration of saline (HU), allopurinol at a dose of 10 mg/kg (HU + ALP), or QALE at a dose of 50 (HU + QA50) or 250 mg/kg (HU + QA250) during 7 days prior to inducing hyperuricemia in mice. The rectangular bars and their error bars represent the means and standard deviations, respectively (*n* = 5); ^*∗*^significantly different from the normal group; ^#^significantly different from the HU group (*p* < 0.05, ANOVA* a posteriori* Tukey's multiple range test).

The effect of QALE on serum uric acid levels in potassium oxonate-induced hyperuricemic mice is shown in Figure 2. Serum uric acid concentrations in the hyperuricemic mice (HU group) at 1 h after intraperitoneal injection of potassium oxonate, a urate oxidase inhibitor, were significantly higher than those in normal mice, indicating that the mouse model of hyperuricemia was successfully established, consistent with a previous report [17]. The serum uric acid concentrations in the HU group were significantly reduced by oral pretreatment of allopurinol (HU + ALP) or QALE at doses of 50 and 250 mg/kg (HU + QA50 and HU + QA250) over 7 days.

### 3.5. In Vivo Antihyperuricemic Effect of QALE on Hepatic and Serum XO Activity

**Figure 3:**
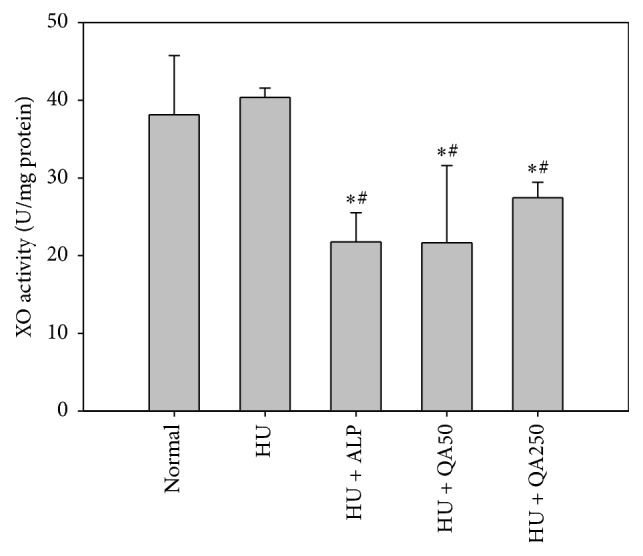
Hepatic xanthine oxidase (XO) activity after oral administration of saline in normal mice (normal) or after oral administration of saline (HU), allopurinol at a dose of 10 mg/kg (HU + ALP), or QALE at a dose of 50 (HU + QA50) or 250 mg/kg (HU + QA250) during 7 days prior to inducing hyperuricemia in mice. The rectangular bars and their error bars represent the means and standard deviations, respectively (*n* = 5); ^*∗*^significantly different from the normal group; ^#^significantly different from the HU group (*p* < 0.05, ANOVA* a posteriori* Tukey's multiple range test).

**Figure 4:**
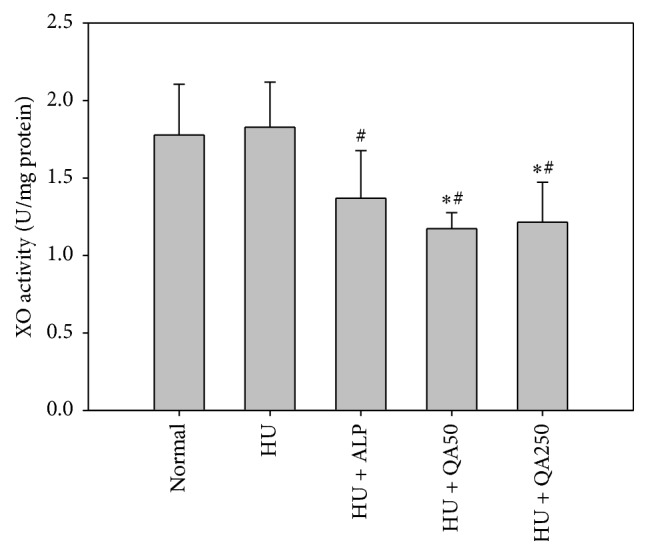
Serum xanthine oxidase (XO) activity after oral administration of saline in normal mice (normal) and after oral administration of saline (HU), allopurinol at a dose of 10 mg/kg (HU + ALP), or QALE at a dose of 50 (HU + QA50) or 250 mg/kg (HU + QA250) during 7 days prior to inducing hyperuricemia in mice. The rectangular bars and their error bars represent the means and standard deviations, respectively (*n* = 5); ^*∗*^significantly different from the normal group; ^#^significantly different from the HU group (*p* < 0.05, ANOVA* a posteriori* Tukey's multiple range test).

The effects of QALE on hepatic and serum XO activity in potassium oxonate-induced hyperuricemic mice are shown in Figures 3 and 4. Hepatic XO activity in hyperuricemic mice was significantly reduced by one week of oral pretreatment of allopurinol (by 46.0%) or QALE at doses of 50 and 250 mg/kg (by 46.3 and 32.0%, resp.), as shown in Figure 3. Similarly, serum XO activity in hyperuricemic mice was significantly reduced by one week of oral pretreatment of allopurinol (by 25.1%) or QALE at doses of 50 and 250 mg/kg (by 35.8 and 33.5%, resp.), as shown in Figure 4. However, there was no significant difference in hepatic or serum XO activity between normal mice and hyperuricemic control mice.

### 3.6. Identification of Active Constituents

**Figure 5:**
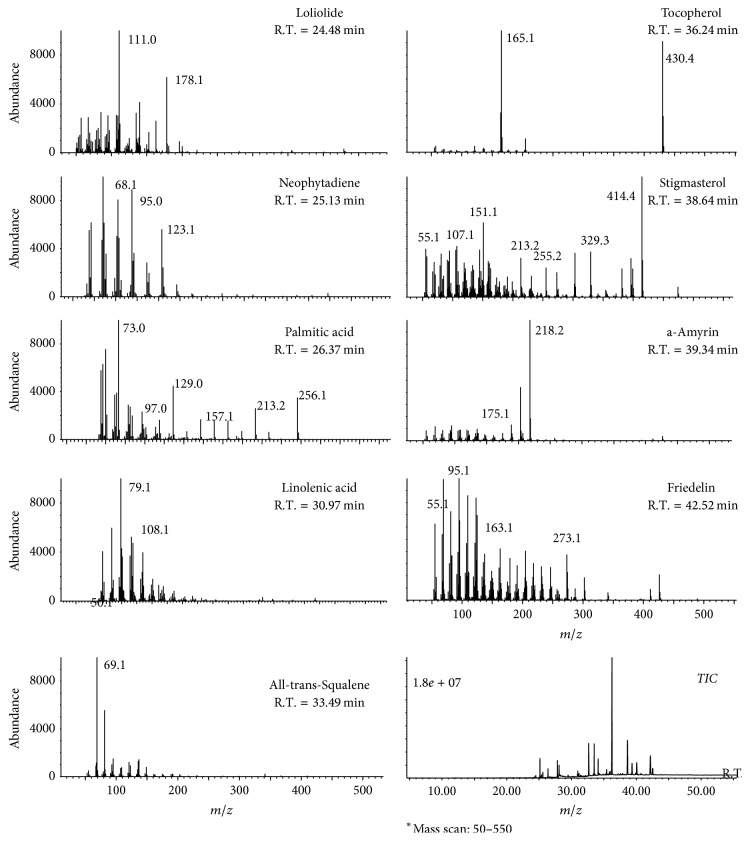
Representative GC-MS chromatogram to show bioactive constituent profiles of QALE.

**Figure 6:**
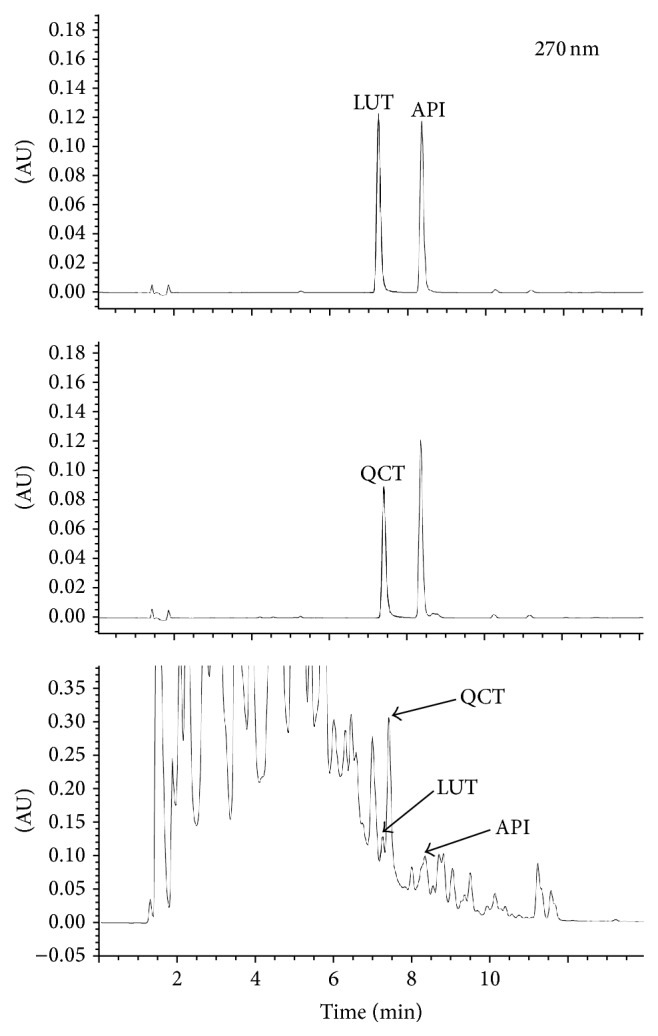
Representative HPLC chromatogram to show bioactive constituent profiles of QALE (API: apigenin; LUT: luteolin; QCT: quercetin).

GC-MS and HPLC analyses were performed to identify active constituents from QALE with antioxidant and XO inhibitory activities. Typical GC-MS and HPLC chromatograms of phytochemical contents and their retention times are shown in Figures 5 and 6. Nine compounds [i.e., vitamin E (28.6%), loliolide (0.38%), neophytadiene (2.36%), stigmasterol (11.2%), palmitic acid (1.55%), *α*-amyrin (3.96%), linolenic acid (0.94%), friedelin (2.97%), and all-trans-squalene (4.13%)] were identified by GC-MS analysis. Additionally, three compounds [i.e., quercetin (0.212%), luteolin (0.0279%), and apigenin (0.0426%)] were identified by HPLC analysis.

## 4. Discussion

This study aimed to determine the antioxidant, XO inhibitory, and antihyperuricemic activities of QALE and identify its active constituents. In our preliminary study, air-dried powder of QA leaf was extracted with various solvents (such as hexane, ethyl acetate, ethanol, and water) and the ethyl acetate extract was found to possess higher XO inhibitory, DPPH free radical scavenging activity, and reducing power than other extracts (data not shown). The ethyl acetate extraction condition was then optimized with respect to its antioxidant and XO inhibitory activity (data not shown). Based on these results, the ethyl acetate extract was selected for further in vitro and in vivo characterization.

Our present results clearly indicate that the QALE is enriched with phenols and flavonoids (Table 2). Polyphenols and flavonoids of natural resources are widely known to possess various biological activities [20, 21]. Several phenols and flavonoids have been shown to possess antioxidant and XO inhibitory activity with ability to decrease serum uric acid levels [21, 22]. Therefore, serum uric acid level and hepatic xanthine oxidase activity were evaluated in this study to determine the antihyperuricemic effect of QALE.

As shown in Figures 2–4, QALE significantly reduced serum uric acid levels and inhibited the activity of hepatic and serum XO even at a relatively low oral dose (50 mg/kg). These results clearly indicate that one week of oral administration with QALE can markedly alleviate the hyperuricemic state in mice. There was no significant difference in hepatic or serum XO activity between the normal mice group and hyperuricemic control mice group, suggesting that intraperitoneal pretreatment with potassium oxonate, a known uricase inhibitor, does not affect the XO activity in mice.

As shown in Figure 5, we identified several bioactive markers related to antioxidant and antihyperuricemic properties, including vitamin E, loliolide, neophytadiene, stigmasterol, palmitic acid, *α*-amyrin, linolenic acid, friedelin, and all-trans-squalene by GC-MS analysis. A previous study using vitamin E-deficient rabbits has reported that deficiency of vitamin E could be responsible for increased accumulation of hepatic XO [23]. Moreover, oral supplementation with palm vitamin E has been reported to be able to reduce gastric XO activity in rats exposed to water-immersion restraint stress [24]. Stigmasterol has also been reported to be able to inhibit hepatic lipid peroxidation and induce catalase and superoxide dismutase activities, suggesting its antioxidant property [25]. *α*-Amyrin has been shown to exert anti-inflammatory effect by reducing the levels of prostaglandin E2 and COX-2 expression in mice with 12-O-tetradecanoylphorbol-acetate- (TPA-) induced skin inflammation [26]. Squalene also possesses antioxidant property with ability to prevent lipid peroxidation in human skin surface exposed to oxidative stress such as sunlight exposure [27]. Friedelin and loliolide are also well known antioxidant agents with scavenging effects on DPPH, nitric oxide, and/or superoxide radicals [28, 29]. Friedelin also has potent suppressive effect on lipid peroxidation [28]. Similarly, palmitic acid has been reported to be able to significantly reduce H_2_O_2_ generation both by neutrophils and in the XO system [30]. Linolenic acid is known to be able to downregulate inducible nitric oxide synthase (iNOS) and cyclooxygenase-2 (COX-2) expression, thereby reducing nitric oxide (NO) and prostaglandin E2 production in lipopolysaccharide- (LPS-) activated RAW264.7 cells with anti-inflammatory activity [31]. Additionally, three known XO inhibitors were further identified in HPLC analysis (Figure 6). Previous studies have reported that luteolin and quercetin can inhibit XO activity in a competitive manner, while apigenin can inhibit it in a mixed manner [17, 32].

Taken together, the present study shows that QALE possesses antioxidant, XO inhibitory, and antihyperuricemic activities. In addition, several phytochemicals identified from the QALE might be responsible for its biological activities. This study provides a good basis for future development of QA-based dietary or medicinal preparations as an alternative to allopurinol.

## 5. Conclusions

The present study investigated the antioxidant and antihyperuricemic effects of QALE and identified major active phytochemicals therein. The ethyl acetate extract of QA leaf was shown to possess potent in vitro antioxidant and XO inhibitory activities. Our in vivo mouse study demonstrated that QALE at a relatively low oral dose (50 mg/kg) could inhibit hepatic XO activity and significantly alleviate hyperuricemia to the extent comparable to allopurinol. Several active compounds including vitamin E known to possess XO inhibitory activity were identified from QALE by GS-MS and HPLC analyses. To the best of our knowledge, this is the first study that reports the active constituents and antihyperuricemic effects of QA. Our results suggest that it might be feasible to use QALE as a food therapy or alternative medicine to alleviate hyperuricemia and gout.

## Figures and Tables

**Table 1:** Analytical conditions of the GC-MS and HPLC methods used in this study.

Parameter	Condition			
*GC-MS*				
Column	Agilent HP-5MS fused silica capillary column (30 m × 0.25 mm *i.d.*, 0.25 *μ*m film thickness)			
Carrier	Helium			
Split	1 : 5			
Injection volume	1 *μ*L			
MS source	230°C			
MS quad	150°C			
Analytical temperature		Rate	Value	Hold time
	Initial		65°C	10 min
	Ramp	10°C/min	300°C	22 min
	Total	55.5 min		
Thermal aux	300°C			
Electron ionization	70 ev			
Mass range	50–550 amu			
Scan method	Full scan			

*HPLC*				
Column	Zorbax extended C18 (C18, 4.6 mm × 150 mm, 5 *µ*m)			
Flow rate	1 mL/min			
Injection volumn	10 *μ*L			
UV detection	270 nm			
Run time	30 min			
Gradient flow	Time (min)	ACN (v/v%)	0.2% phosphoric acid (v/v%)	
	0	15	85	
	10	15	85	
	15	75	25	
	20	75	25	
	25	100	0	
	26	15	85	
	30	15	85	

**Table 2:** Antioxidant activity and total contents of phenols and flavonoids of QALE.

Parameter	QALE	Ascorbic acid
DPPH IC_50_ (*μ*g/mL)	10.6 ± 0.5	6.95 ± 0.30
Reducing power (Ascorbic acid eq. *μ*g/50 *μ*g ext.)	36.3 ± 3.5	
Total phenol (mg/g ext.)	126 ± 2	
Total flavonoid (mg/g ext.)	27.6 ± 1.4	

## Abbreviations

QA: * Quercus acuta* Thunb.
QALE: QA leaf extract
XO: Xanthine oxidase.
